# Blinded, randomized, sham-controlled clinical trial assessing the efficacy of a novel piezoelectric extracorporeal shockwave device following TPLO

**DOI:** 10.3389/fvets.2025.1600116

**Published:** 2025-07-24

**Authors:** Shannon L. Campbell, Ashley L. Franklin, Samuel P. Franklin

**Affiliations:** Kansas City Canine Orthopedics, Shawnee, KS, United States

**Keywords:** canine (dog), cranial cruciate ligament (CCL), objective gait analysis, extracorporeal shockwave, TPLO

## Abstract

**Introduction:**

Extracorporeal shockwave therapy (ESWT) devices have been used to effectively treat a wide variety of musculoskeletal conditions in veterinary medicine. However, several factors have limited ESWT device adoption, including that they are commonly loud, uncomfortable, and application typically requires sedation or anesthesia. A novel piezoelectric ESWT device has been developed which is lightweight, quiet, and does not require sedation for application. The purpose of this study is to assess the safety and efficacy of this novel device in clinical application.

**Materials and methods:**

This was a prospective blinded trial in which dogs were randomly allocated to receive three ESWT treatments, or three sham treatments, immediately following and at 2 and 4 weeks post tibial plateau leveling osteotomy (TPLO). The primary outcome measure was clinical function at 4 and 8 weeks post-operatively based on objective gait analysis. Secondary outcome measures included owner subjective assessments (Canine Brief Pain Inventory and Liverpool Osteoarthritis for Dogs questionnaires) at 2, 4, and 8 weeks post-operatively and radiographic assessment of patella tendon thickness and osteotomy healing at 8 weeks post-operatively.

**Results:**

All dogs completed all ESWT treatments without the need for sedation and no adverse events associated with ESWT use were observed or reported. Dogs in the ESWT group bore significantly more weight at a trot at 4 weeks post-operatively in comparison to dogs in the sham treatment group. There were no statistically significant differences between groups based on owner subjective assessments or radiographic assessments.

**Discussion:**

The study demonstrated that the device was safe and that treatments could be completed without the need for sedation. Subjective owner assessment and radiographic bone healing were not significantly improved with treatment. However, the primary outcome measure, objective gait analysis using a pressure sensitive walkway, showed that dogs in the treatment group bore significantly more weight 4 weeks post-operatively at a trot in comparison to sham-treated dogs.

## Introduction

Extracorporeal shockwave therapy (ESWT) devices have been evaluated for several musculoskeletal conditions in dogs (1–5). While positive results have been reported (3, 6, 7), application has been limited to some extent because commonly investigated shockwave generators are large and loud, administration of energy to the patient is usually uncomfortable, and sedation is commonly needed for application (1–9). Improvement of ESWT devices that enables consistent application without the need for sedation or anesthesia, with retained efficacy, would likely be more widely applied. It would also be ideal if such units can be quieter, lighter, and more easily portable.

To address these current limitations, a novel piezoelectric device has been developed that incorporates fifteen piezoceramic plates which are mechanically focused, electrically time aligned, and quickly energized to repeatedly and consistently produce a focused shockwave. Interchangeable standoffs allow for the selection of the focal depths of energy delivery ranging from 2 mm to 30 mm. Total therapeutic depth, defined by locations at which energy exceeds 5 MPa, can reach from 2 mm to 60 mm depth depending upon the standoff used. The maximum energy flux density produced by the device is 0.22 mj/mm^2^. According to the manufacturer, this method of shockwave creation is repeatable and durable, and the device can generate 5 million shocks prior to needed re-calibration or servicing. The unit is battery-powered and weighs 0.4 kg (0.88 lbs). It also has been used by the authors in a pilot study in which 5 dogs undergoing tibial plateau leveling osteotomy (TPLO) were treated with the device immediately post-operatively and at 2 weeks post-operatively. All dogs were assessed by the veterinarian, canine brief pain inventory (10), client diaries for recording of adverse events, and radiographs up to 2 months post-operatively. The ESWT treatment was well-tolerated by all patients without the need for sedation and no adverse events were observed by the veterinarian or reported by the owners. While this novel piezoelectric device is appealing because of these aforementioned characteristics, the salient question is whether this device results in a measurable therapeutic benefit.

The purpose of this clinical trial was to test the efficacy of ESWT following TPLO when using this novel piezoelectric device. More specifically, the study was designed to assess the safety and efficacy of such ESWT in improving bone healing, mitigating patella tendon thickening, and improving weight bearing. Consistent with the unpublished pilot study, we hypothesized that the device would be safe and its application well-tolerated without the need for sedation to complete the treatments. Further, the primary outcome measure in this study was objective gait analysis using a pressure sensitive walkway and we hypothesized that the use of ESWT would result in significantly improved weight bearing as assessed using such objective gait analysis. A second hypothesis was that patella tendon thickness, as measured radiographically, would be less in dogs treated by ESWT than control dogs. The final hypothesis was that the radiographic assessment of bone healing would not be statistically significantly different between the two groups.

## Materials and methods

### Patient eligibility and enrollment

Dogs were eligible for inclusion if they were between 1 and 10 years of age, 16 kg and 60 kg body weight, and could be appropriately treated with a 3.5 mm small, standard, broad, or jumbo sized TPLO plate. Exclusion criteria included the use of ESWT or photobiomodulation during the 2 months preceding enrollment, use of bedinvetmab (Librela; Zoetis Inc, Kalamazoo, MI), use of polysulfated glycosaminoglycan supplementation (Adequan; American Regent Inc, Shirley, NY) in the 6 weeks prior to enrollment, or previous intra-articular joint injections. All dogs needed to have ceased receiving any oral analgesics such as gabapentin or oral non-steroidal anti-inflammatory drugs (NSAIDs) at least 2 days prior to enrollment and acquisition of baseline data. Exclusion criteria also included any systemic illness, neurologic disease, or musculoskeletal pathology, such as contralateral CCL disease, as based upon physical examination or radiography. Dogs that had the contralateral stifle treated with a TPLO a minimum of 6 months prior were eligible for enrollment.

### Subjective owner assessments

Owners completed both the canine brief pain inventory (CBPI) (10) and liverpool osteoarthritis for dogs (LOAD) (11) questionnaire pre-operatively and at 2, 4 and 8 weeks post-operatively. For consistency, the same owner completed the surveys at each visit. If the owner who originally completed the surveys was unavailable to attend the recheck appointment, the surveys were emailed to the participating owner to be filled out within 48 h of the recheck appointment.

### Objective gait analyses

Gait analysis for each patient was performed on the day of surgery (preceding surgery) and at 4 and 8 weeks post-operatively. Gait data collection was performed by the same handler (SLC), using a GAITFour pressure sensitive walkway system (12) (Gait4Dog, CIR Systems Inc., Franklin, NJ). Cameras capturing video of each trial were placed at both ends of the walkway system to assess position of the head and body in both directions of travel. Gait data were collected consecutively until a minimum of 5, valid-appearing trials at a walk, and 5 valid-appearing trials at trot, were completed. Processing of the gait data was subsequently performed after the appointment was completed. Gait trials were ultimately considered valid if video footage included the patient moving toward a camera and such video confirmed the head and body were forward facing with no turning of the head and no pulling on the leash for the entirety of the trial. In addition, in order to be considered valid, the velocity needed to have <10% variation and at least 3 complete gait cycles. If the dog was non-weight bearing for the trial a value of 0 was recorded. Once processing was complete, the total pressure index (TPI) for the affected hindlimb from each valid trial as well as the velocity were exported into an excel spreadsheet.

### Surgical technique

All patients were premedicated with hydromorphone (0.1 mg/kg intramuscular) and acepromazine (0.05 mg/kg intramuscular) with propofol (4 mg/kg intravenous) given to effect as the induction agent. The protocol was changed for the last patient due to supply issues when morphine (0.5 mg/kg intramuscular) was used as the premedication agent in place of hydromorphone. All patients received peri-operative cefazolin (22 mg/kg intravenously) given at the time of anesthetic induction and every 90 min thereafter until inhalant anesthesia was discontinued. The anesthesia for all patients was maintained using isoflurane. Each patient received a femoral and sciatic nerve block consisting of bupivacaine (1.5 mg/kg, 50 mg/ml) and dexmedetomidine (0.001 mg/kg; 0.5 mg/ml) prior to surgery using a nerve stimulator. The bupivacaine and dexmedetomidine were mixed and half the total volume administered to the femoral nerve and half to the sciatic nerve. Pre-operative orthogonal radiographs of the stifle were taken under anesthesia for surgical planning purposes. Standard subpatellar arthroscopic portals were made medial and lateral to the patella tendon. The stifle joint of each patient was evaluated with a 2.5 or 2.7 mm 30° arthroscope. The cranial cruciate ligament (CCL) was classified as a complete rupture, a partial incompetent rupture, or a partial competent rupture. Completely ruptured and partial incompetent ruptures were completely debrided and lumped together as “complete ruptures” for the purposes of stratification (see below). Partial competent CCL tears were left intact with debridement of the torn fibers only. The medial meniscus was probed and assessed for pathology. If the menisci were normal they were left untreated. If there was meniscal pathology that necessitated meniscectomy, either partial meniscectomy plus meniscal release, hemi-meniscectomy, or full thickness meniscal release at the caudomedial meniscotibial ligament was performed. Hence, all stifles could be accurately classified as having an intact and normal meniscus or effectively being “meniscal deficient”. A TPLO was then performed using an approach to the proximomedial tibia with elevation of the pes anserinus muscle group. Limited elevation of the popliteus muscle caudally was performed, but no gauze or other material was placed caudally. No elevation of the craniolateral musculature was performed. A jig was not applied. Templating and performance of the TPLO was performed with the center of the osteotomy located at the base of the intercondylar eminences. Rotation of the proximal segment was performed using a rotational pin and with a target final tibial plateau angle of 5°. All patients had locking TPLO plates applied using a combination of locking screws applied proximal to the osteotomy and non-locking screws applied distal to the osteotomy. Closure of the caudal belly of the sartorius muscle often included advancement and placement of one or two suture passes through the patella tendon for security of the closure. Subcutaneous and skin closure was routine.

### Stratification and randomization

Immediately following arthroscopy dogs were allocated to one of three different strata based upon the status of the cranial cruciate ligament and meniscus: (A) complete CCL deficiency with an intact medial meniscus, (B) complete CCL deficiency and meniscal deficiency, or (C) partial competent CCL rupture with an intact medial meniscus; there were no dogs with a partial competent CCL rupture and meniscal deficiency. Within each stratum patients were randomly allocated to either ESWT or sham treatment based on a randomization order created using a randomization tool (Sealed Envelope Ltd. 2024. Create a blocked randomization list. Available from: https://www.sealedenvelope.com/simple-randomiser/v1/lists).

### ESWT treatment

**Figure 1:**
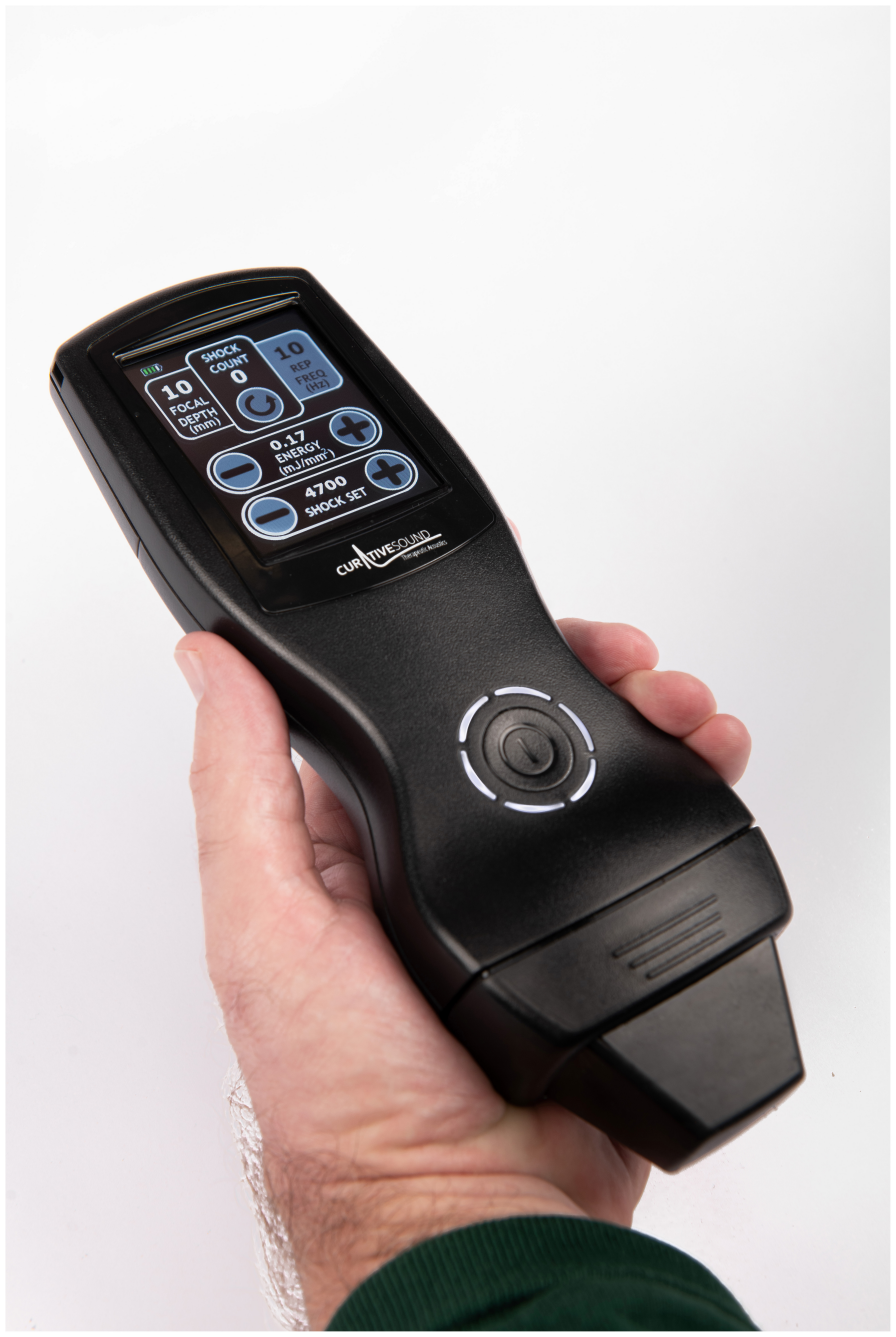
Novel piezoelectric extracorporeal shockwave device.

Immediately following surgery and acquisition of post-operative radiographs, ESWT, or sham treatment, was performed using the novel piezoelectric shockwave device (CS-Pro VET device, Curative Sound, Carmel, IN; Figure 1) with the patient under anesthesia. Treatment included 5 areas: the craniomedial and caudomedial proximal tibia just cranial to and just caudal to the TPLO plate, the craniolateral and caudolateral proximal tibia, and the patellar tendon from the distal patella to the level of insertion on the tibial tuberosity. The 4 lateral and medial areas of the proximal tibia received 1,200 shockwaves per area and the patellar tendon received 2,400 shockwaves. A pre-sterilized standoff, attached to the ESWT device by magnetism, was used in each area to focus the depth of the shockwave. The standoff for the medial areas was 10 mm with an energy flux density of 0.17 mj/mm^2^, the standoff for the lateral areas was 30 mm with an energy flux density of 0.22 mj/mm^2^ and the patellar tendon standoff was 5 mm with an energy flux density of 0.14 mj/mm^2^. The device with the appropriate standoff was moved in small circles over the region of interest and ultrasound gel was used to facilitate shockwave transmission. Dogs in the sham treatment group received the same protocol without the battery included in the device. All treatments were performed by the same investigator (SLC).

Extracorporeal shockwave treatment, or sham treatment, was repeated at the 2 and 4 week recheck after suture removal or gait analysis were performed, respectively. Dogs were not sedated for treatment. At the 4 week recheck, although potentially unnecessary for adequate energy transmission, each patient was re-shaved at the level of the proximal tibia and patella tendon to maintain consistency among all patients in terms of a lack of hair.

### Postoperative radiographs

Orthogonal radiographs were made 8 weeks post-operatively under sedation (dexmedetomidine 0.005 mg/kg and butorphanol 0.1 mg/kg). Following completion of the last 8-week recheck, all radiographs made immediately pre-operatively and at 8 weeks post-operatively were anonymized and then evaluated by one investigator (SPF) blinded to the treatment group. Assessment for bone healing was made based upon the 8 week radiographs using a modified radiographic union scale for tibial fractures (mRUST) that has been assessed for validity in scoring tibial healing following TPLO (13). Patella tendon thickness was measured immediately pre-operatively and on the 8 week recheck radiographs. Thickness was measured at the distal quarter of the tendon's length as this was the one location in which a significant difference was previously identified following ESWT in conjunction with TPLO (3).

### Postoperative care and adverse events monitoring

All patients received post-operative analgesia consisting of carprofen (2.2 mg/kg twice daily orally) and gabapentin (10 mg/kg three times daily orally) each given for 2 weeks. A 7-day course of antibiotics (cephalexin 22 mg/kg three times daily orally) was prescribed as well as trazodone (4–8 mg/kg up to 3 times daily orally) for sedation which could be administered as needed throughout the 8 week recovery period. Indoor activity restriction recommendations included confinement to a designated safe room, pen, crate, or on-leash in the home during the first 8 weeks of recovery. Recommendations for outdoor activity involved a slow increase in leashed walks over 8 weeks beginning with 5–10 min walks during weeks 1–4 and 15–20 min walks during weeks 5–8. Physical rehabilitation was prohibited until patients completed the study.

Diaries were provided to owners for the recording of medication usage and adverse events on the day of surgery. That diary was collected at the 2 week recheck and a new diary was issued; this process was performed iteratively at each recheck until the end of the study. Owners were also contacted by phone at weeks 1, 3 and 7 post-operatively to discuss activity levels and inquire if there had been any adverse events.

### Statistical analyses

A linear mixed model was used to analyze the TPI obtained using the pressure sensitive walkway. The 5 walks and 5 trots with the smallest range in velocity (for each gait) were selected and included in the analysis based on recommendations to use narrow velocity ranges with limited/controlled acceleration in order to minimize data variance (14). The statistical model included fixed factors for treatment, time, strata, walk/trot, and a random intercept for dog. Additionally, the model for TPI included all two- and three-way interactions and one four-way interaction. Linear mixed models were also used to analyze LOAD and CBPI scores with fixed factors for treatment, time, strata and all two and one three-way interaction as well as a baseline covariate and a random intercept for dog.

A Mann-Whitney test was used to compare mRUST scores between treatment groups. An analysis of covariance was used to compare patella tendon thickness between groups with factors for treatment, strata and treatment by strata interaction and a covariate for baseline patella tendon thickness. *p*-values of < 0.05 were considered statistically significant.

## Results

Thirty-two dogs were enrolled in the study with 30 completing the study; one dog was excluded after developing lymphoma approximately 4 weeks after enrollment and one dog was not returned for any follow-up after surgery; all data from those two dogs were excluded. Two additional dogs had minor complications and their TPI, LOAD, and CBPI data from the 8 week recheck only were excluded. Specifically, one dog was allowed off leash and unsupervised for several hours shortly before the 8 week recheck and went from sound to acutely lame just prior to that recheck. Radiographs confirmed a small chip fracture on the distal pole of the patella. The second dog jumped out of an elevated truck immediately prior to entering the hospital at 8 weeks and immediately became lame just prior to such recheck. Both of these dogs were in the ESWT treatment group and all other data from these 2 patients were included. No other complications or adverse events were noted including no adverse events associated with use of the device and provision of the ESWT treatment. All subjects in the treatment group tolerated the use of the shockwave without need for sedation.

The data on age, weight, and gender by treatment group is shown in Table 1. There were 15 dogs each in the ESWT and sham treatment groups. There were 10 dogs in the complete CCL rupture/intact meniscus stratum, nine in the complete CCL rupture and meniscal deficient stratum, and 11 in the partial CCL rupture/intact meniscus stratum.

**Table 1:** Demographic data on included study patients. Note that all dogs were spayed/neutered.

**Group**	**Demo-graphic**	***N***	**Mean**	**Std dev**	**Min**	**Max**
ESWT	Age (years)	15	6.1	1.5	4.0	9.0
	Weight (kg)	15	33.5	7.0	22.9	45.5
	Female	8				
	Male	7				
Sham	Age	15	5.7	1.7	3.0	9.0
	Weight	15	31.8	8.2	20.4	45.3
	Female	11				
	Male	4				
All	Age	30	5.9	1.6	3.0	9.0
	Weight	30	32.6	7.6	20.4	45.5
	Female	19				
	Male	11				

### Objective gait analysis and TPI

**Figure 2:**
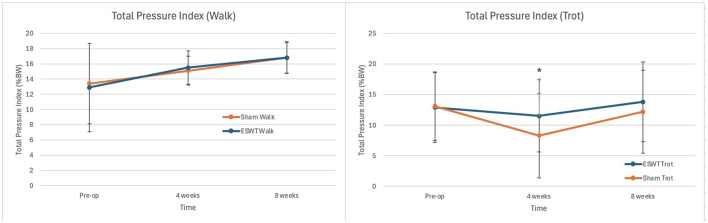
Total pressure index (TPI, +/– standard deviation) at 0, 4, and 8 weeks post-operatively. **(Left)** Mean TPI at Walk. **(Right)** Mean TPI at Trot. *Significance found at 4 weeks at a trot, *p* = 0.04. *p*-values at all other times points were >0.05.

There was a statistically significant difference in TPI between the ESWT and sham treatment groups at a trot 4 weeks post-operatively with greater weight bearing in the ESWT group (*p* = 0.04). This difference in weight bearing at a trot was no longer statistically significant by 8 weeks post-operatively. There were no statistically significant differences at 4 and 8 weeks post-operatively at a walk. Data on TPIs are shown in Figure 2.

### Subjective owner assessments

#### CBPI

**Figure 3:**
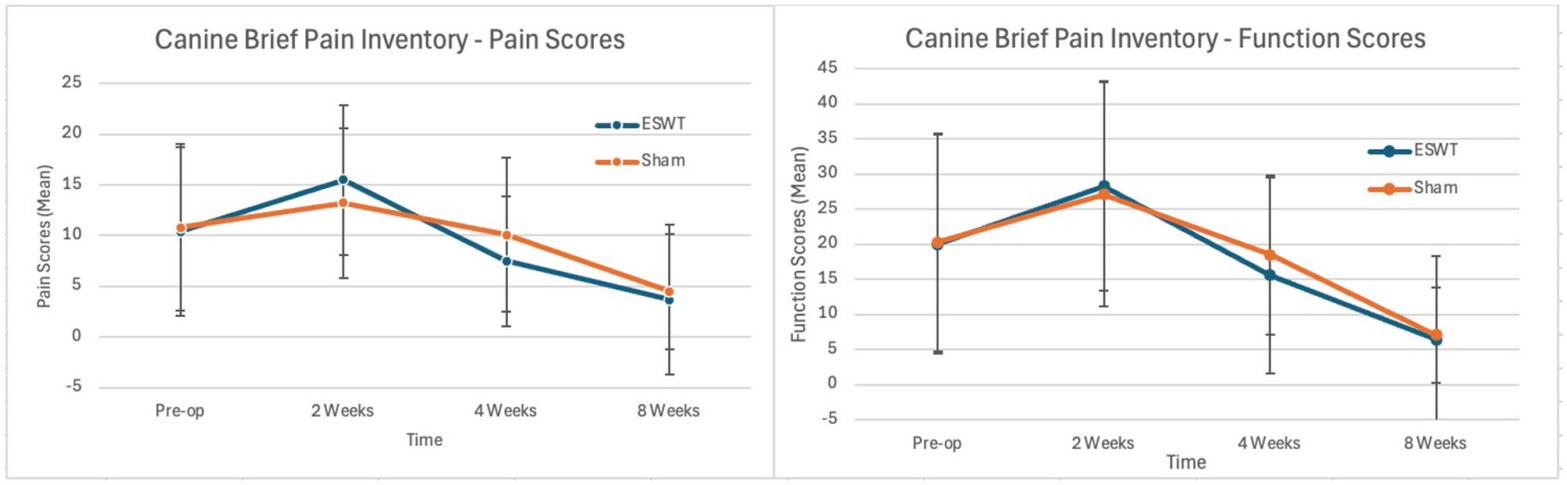
Canine brief pain inventory (CBPI, +/– standard deviation) scores pre-operatively and at 2, 4, and 8 weeks post-operatively. **(Left)** Mean pain scores over time. **(Right)** Mean function scores over time. All *p*-values > 0.05. Lower scores indicate less pain and better function.

There were no statistically significant differences in either the pain or function scores between the two groups at 2, 4, or 8 weeks post-operatively. The CBPI scores are shown in Figure 3.

#### LOAD

**Figure 4:**
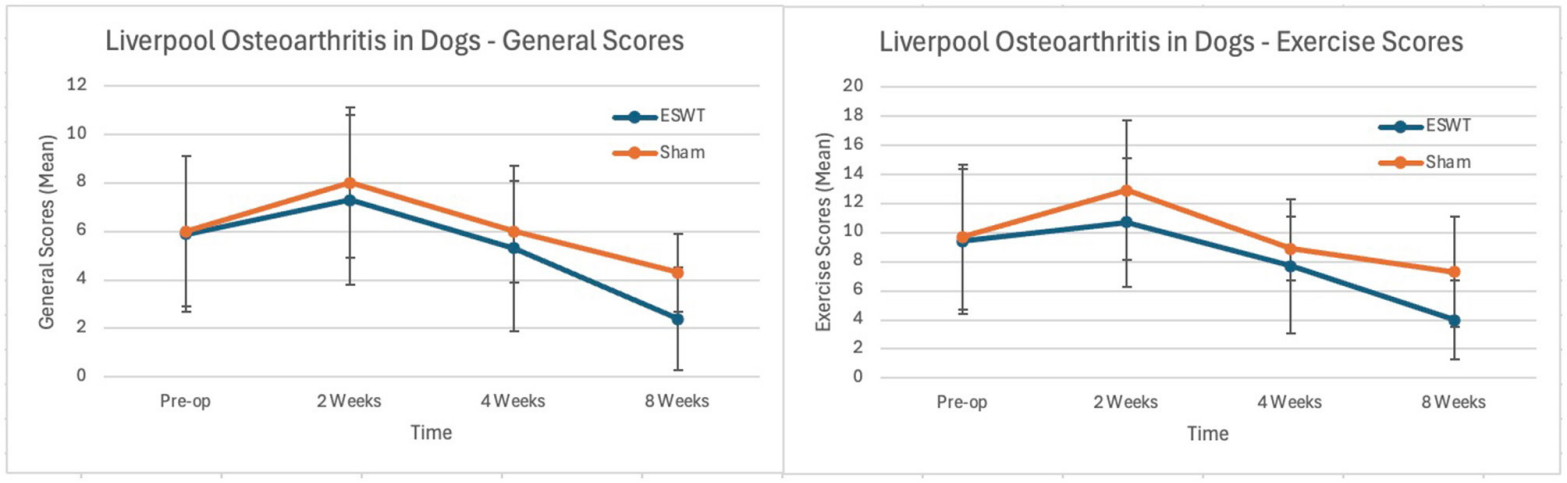
Scores from pre-operative and 2, 4, and 8 weeks post-operative Liverpool Osteoarthritis in Dogs (LOAD) surveys (+/– standard deviation). **(Left)** Mean general mobility scores. **(Right)** Mean exercise scores. All *p*-values > 0.05. Lower scores indicate greater mobility.

There were no statistically significant differences in either the general mobility or mobility with exercise scores between the two groups at 2, 4, or 8 weeks post-operatively. The LOAD scores are shown in Figure 4.

### Radiographic assessments

There were no statistically significant effects of ESWT treatment on bone healing scores (*p* = 0.34) or measures of patella tendon thickness (*p* = 0.36). The mRUST scores and measures of patella tendon thickness are shown in Tables 2, 3 below respectively.

**Table 2:** Bone healing scores: descriptive statistics and *p*-value.

**Bone healing mRUST score**							
**Treatment group**	***N***	**Median**	**Lower quartile**	**Upper quartile**	**Minimum**	**Maximum**	***p*** **-value**
ESWT	15	9.0	7.0	10.0	6.0	11.0	0.34
Sham	15	9.0	7.0	11.0	7.0	12.0	

**Table 3:** Patella tendon scores: descriptive statistics and *p*-value.

**Treatment group**		***N***	**Mean**	**Std dev**	**Minimum**	**Maximum**	***p*-value**
ESWT	Pre-op	15	3.3	0.7	2.2	5.1	0.36
	Post-op	15	7.1	1.8	4.1	9.9	
Sham	Pre-op	15	3.4	0.7	2.7	5.0	
	Post-op	15	6.6	1.2	4.7	9.3	

## Discussion

The study was completed with no adverse effects noted by owners or during patient physical examinations. In addition, all applications of ESWT in the post-operative period were well tolerated and completed without the need for sedation. Although these findings were expected based upon the unpublished pilot study conducted previously, they do stand in contrast to previous studies assessing electrohydraulic ESWT following TPLO in which either sedation or anesthesia were used (3, 6, 7). These findings are important given the broader goal of making ESWT more broadly applicable by improving patient tolerability of its application.

The primary goal of the study was to determine whether treatment with this novel piezoelectric device would be sufficient to result in a clinical improvement based upon objective gait analysis. The data obtained in this study showed that dogs treated by this novel ESWT device bore significantly more weight at a trot at 4 weeks post-operatively in comparison to sham treated dogs. Given that kinetic objective gait analysis is the gold standard outcome measure in research studies of dogs with musculoskeletal disease (14), and is not subject to caregiver placebo effect (15), we conclude that the energy provided by this device is sufficient to produce improvement. Further, these data are consistent with a previous study using an electrohydraulic device which also showed superior weight bearing in dogs treated with ESWT following TPLO based upon force plate analysis (7). Hence, the consistency of the data support the conclusion that ESWT, whether provided with the aforementioned electrohydraulic device or the novel piezoelectric used in this study, can result in improved weight bearing during recovery from TPLO.

While we detected a significant difference between treatment groups based upon the primary outcome measure, we failed to detect a statistically significant difference between the sham and ESWT treatment groups in the secondary outcome measures including owner subjective assessments. The lack of detectable difference in the owner's subjective assessments may be because there truly is no difference between the two groups, or the results may be attributable to type II statistical error. The scores for the ESWT group were numerically superior, but not statistically significant, in ten of the 12 post-operative assessments using this methodology (ie six LOAD comparisons and six CBPI comparisons). Three previous studies assessing electrohydraulic ESWT following TPLO did not assess LOAD or CBPI scores so we cannot compare our results to previous studies on ESWT in conjunction with TPLO (3, 6, 7).

The data did not show a significant decrease in patella tendon thickening in dogs in the ESWT treatment group. This result contrasts with what we had hypothesized and with a previous study that shows mitigation in patella tendon thickening following use of electrohydraulic ESWT (3). The explanation for this difference is unclear and there are numerous possibilities that include lack of a true difference with the ESWT provided or a type II statistical error. An additional potential explanation is that there was no difference that was in part attributable to our surgical technique. The surgeon in this study (SPF) commonly incorporates one or two suture passes through the patella tendon when advancing and securing the caudal belly of the sartorius muscle during closure. Placement of suture (poliglecaprone) in the patella tendon that would still be present at 8 weeks post-operatively might perpetuate inflammation of the tendon and peritendinous tissues and mitigate ability of ESWT to provide a measurable difference. Suturing to the patella tendon is not done by all surgeons and would ideally be specified in future studies assessing the efficacy of new technologies aiming to ameliorate patella tendon thickening post-TPLO surgery.

Finally, the data did not show a significant difference between the sham and ESWT treatment groups with regard to bone healing. This result was not surprising and was consistent with our *a priori* hypothesis that we would not detect a difference in this outcome measure. TPLO is a difficult model to assess treatments that may hasten bone healing, likely because this osteotomy is well apposed, rigidly stable, and heals consistently well over an 8–12 week time frame in the vast majority of dogs (16). Consequently, we do not conclude broadly that ESWT, and with this device specifically, cannot improve bone healing. Rather, we conclude that the ESWT provided in this study failed to result in significant acceleration of bone healing following TPLO, and such lack of difference is potentially attributable to difficulty in detecting a difference given the successful progression in bone healing of all patients in the study.

There were numerous limitations of this study, one being a sample size of 30 dogs. Although this number of dogs is not small by standards in veterinary clinical trials, it is in general a relatively small number of patients. In turn, this may have hampered our ability to detect significant differences, particularly in subjective owner assessment and bone healing. Other limitations include our assessment of the radiographic outcomes by a single observer, rather than with multiple observers and assessment of repeatability. We doubt that this precluded detection of a significant benefit as previous studies have assessed repeatability of these outcome measures. An additional limitation of these data is that this study does not involve a direct comparison between different extracorporeal shockwave devices and so we cannot draw any conclusions regarding the inferiority or superiority of this device in terms of efficacy in comparison to any other extra corporeal shockwave devices.

In general, a high percentage of dogs with cranial cruciate ligament disease treated by TPLO recover and have a clinically successful outcome. Consequently, while treatments that enhance healing and recovery following TPLO are appealing, they are rarely necessary in order to achieve good clinical results. Accordingly, the purpose of this study was not motivated by a critical need to find a solution to recovery following TPLO, but rather to assess whether this novel piezoelectric shockwave device can produce a measurable benefit while being safe (based on the outcomes assessed up to 8 weeks) and well tolerated without the need for sedation. Based upon objective gait analysis, we conclude that treatment with this novel shockwave device can provide a clinically detectable benefit with improved weight bearing at 4 weeks post TPLO in comparison to sham treated dogs. There were no statistically significant differences in subjective owner assessment, patella tendon thickening, or bone healing. There were also no complications or adverse events associated with the ESWT. This study provides a proof of principle that treatment with this novel device can result in clinical improvement and is safe. Hence, further studies are warranted to determine for which other conditions ESWT using this device could be beneficial and this device could potentially be compared directly to other ESWT devices.

## Data Availability

The datasets presented in this article are not readily available but the data can be requested from Curative Sound LLC. Requests to access the datasets should be directed to https://curative-sound.com.
